# Morbidity and mortality in schizophrenia with comorbid substance use disorders in Finland and Sweden

**DOI:** 10.1192/j.eurpsy.2021.636

**Published:** 2021-08-13

**Authors:** M. Lähteenvuo, A. Batalla, J. Luykx, E. Mittendorfer-Rutz, A. Tanskanen, J. Tiihonen, H. Taipale

**Affiliations:** 1 Department Of Forensic Psychiatry, Niuvanniemi hospital, Kuopio, Finland; 2 Psychiatry, University Medical Centre Utrecht, Utrecht, Netherlands; 3 Department Of Psychiatry, Utrecht University, Utrecht, Netherlands; 4 Department Of Clinical Neuroscience, Karolinska Institutet, Stockholm, Sweden

**Keywords:** schizophrénia, substance abuse disorders, epidemiology, dual diagnosis

## Abstract

**Introduction:**

Schizophrenia is highly comorbid with substance use disorders (SUD) but large epidemiological cohorts exploring the prevalence and prognostic significance of SUD are lacking.

**Objectives:**

To investigate the prevalence of SUD in patients with schizophrenia in Finland and Sweden, and the effect of these co-occurring disorders on risks of psychiatric hospitalization and mortality.

**Methods:**

45,476 individuals with schizophrenia from two independent national cohort studies, aged <46 years at cohort entry, were followed during 22 (1996-2017, Finland) and 11 years (2006-2016, Sweden). We first assessed SUD prevalence (excluding smoking). Then we performed Cox regression on risk of psychiatric hospitalization and mortality in patients with schizohrenia and SUD compared with those without SUD.

**Results:**

The prevalence of SUD in specialized healthcare ranged from 26% (Finland) to 31% (Sweden). Multiple drug use and alcohol use disorders were the most prevalent SUD, followed by cannabis use disorders. Any SUD comorbidity, and particularly multiple drug use and alcohol use, were associated with 50% to 100% increases in hospitalization and mortality compared to individuals without SUD. Elevated mortality risks were observed especially for deaths due to suicide and other external causes. All results were similar across countries.

**Conclusions:**

Co-occurring SUD, and particularly alcohol and multiple drug use, are associated with high rates of hospitalization and mortality in patients with schizophrenia. Preventive interventions should prioritize detection and tailored treatments for these co-morbidities, which often remain underdiagnosed and untreated.

**Conflict of interest:**

ML: Genomi Solutions Ltd, Nursie Health Ltd, Sunovion, Orion Pharma, Janssen-Cilag, Finnish Medical Foundation, Emil Aaltonen Foundation. HT, EMR, AT: Eli Lilly, Janssen–Cilag. JT: Eli Lilly, Janssen-Cilag, Lundbeck, Otsuka.

